# Design and Construction of a Single-Tube, LATE-PCR, Multiplex Endpoint Assay with Lights-On/Lights-Off Probes for the Detection of Pathogens Associated with Sepsis

**DOI:** 10.1155/2012/424808

**Published:** 2012-12-26

**Authors:** Rachel K. Carver-Brown, Arthur H. Reis, Lisa M. Rice, John W. Czajka, Lawrence J. Wangh

**Affiliations:** ^1^Department of Biology, Brandeis University, Waltham, MA 02453-2728, USA; ^2^Smiths Detection Diagnostics, Edgewood, MD 21040, USA

## Abstract

*Aims*. The goal of this study was to construct a single tube molecular diagnostic multiplex assay for the detection of microbial pathogens commonly associated with septicemia, using LATE-PCR and Lights-On/Lights-Off probe technology. *Methods and Results*. The assay described here identified pathogens associated with sepsis by amplification and analysis of the 16S ribosomal DNA gene sequence for bacteria and specific gene sequences for fungi. A sequence from an unidentified gene in *Lactococcus lactis* subsp. *cremoris* served as a positive control for assay function. LATE-PCR was used to generate single-stranded amplicons that were then analyzed at endpoint over a wide temperature range in a specific fluorescent color. Each bacterial target was identified by its pattern of hybridization to Lights-On/Lights-Off probes derived from molecular beacons. Complex mixtures of targets were also detected. *Conclusions*. All microbial targets were identified in samples containing low starting copy numbers of pathogen genomic DNA, both as individual targets and in complex mixtures. *Significance and Impact of the Study*. This assay uses new technology to achieve an advance in the field of molecular diagnostics: a single-tube multiplex assay for identification of pathogens commonly associated with sepsis.

## 1. Introduction

Globally, sepsis affects millions of people, killing at least 1 in 4 [1, 2]. Nucleic-acid-testing- (NAT-) based methods of pathogen identification have for some time been regarded as a desirable alternative to conventional culture-based diagnostic methods for analysis of septicemia specimens [3–23]. Early goal directed therapy within the first 3 hours of the clinical presentation of severe sepsis and septic shock has significantly improved outcomes [1, 24–31]. The use of molecular diagnostic methods is logical because they are rapid, sensitive, specific, and reproducible [32]. 

We have optimized linear-after-the-exponential PCR (LATE-PCR) to overcome the challenges inherent to construction of a highly informative single-tube multiplex assay for septicemia where an unlimited number of possible pathogens must be detected. LATE-PCR is a form of non-symmetric PCR that generates single-stranded DNA making it possible to probe and quantify these strands at endpoint [33–41]. Utilizing both the expanded temperature and fluorescent space now available for detection of targets at endpoint, LATE-PCR assays can readily be multiplexed [40]. 

Two conceptually distinct sepsis single tube multiplex assays have been designed and tested in our laboratory. One design used a combination of probes and primers specific to selected genes for each target to differentiate 17 pathogens of interest as well as an internal control [42]. The second design, described here, amplified just three targets, a bacterial 16S rDNA sequence, a sequence in the *Candida* P450L1A1 gene, and a sequence in *Lactococcus lactis* subsp. *cremoris* that served as an internal control. This conceptually simpler assay distinguished different possible bacterial species by virtue of the fact that the target was analyzed at endpoint using a single colored set of Lights-On/Lights-Off probes. Lights-On/Lights-Off probes are a recently described technology that makes it possible to readily distinguish sequence variants among the single-stranded products generated by a LATE-PCR multiplexed reaction [43]. The *Candida* species, as well as the *L. lactis* control were distinguished through the use of gene specific probes also analyzed at endpoint in different colors.

## 2. Materials and Methods

### 2.1. 16S Sepsis Assay Configuration and Parameters

**Figure 1:**
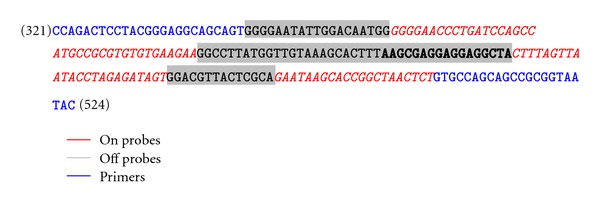
On/Off Probe placement for the 321–524 bp region of the 16S rDNA gene of *A. baumannii* (NC_010400). The On probes are in red text italics and Off probes highlighted in grey. The primers are in blue text. Distinctions between two adjacent probes are in bold.

The 16S sepsis multiplex assay design is shown in Table 1. In this design the 16S bacterial genomic DNA was detected with the Lights-On/Lights-Off probes in the Quasar 670 fluorescent dye channel while the *Lactococcus lactis* subsp. *cremoris* control DNA and *Candida* species of interest were detected in the Cal Org 560 and Cal Red 610 channels, respectively.  Figure 1 shows the placement of the primers and Lights-On/Lights-Off probes in the 16S region of choice. The Lights-Off probe locations are highlighted in grey while the Lights-On probe locations are highlighted in red. The full region of 157 bases between the primers was completely covered with Lights-On/Lights-Off probes. The predicted melting temperatures, Tms, of the Lights-On/Lights-Off probes are shown in Table 2.

Table 3 lists the sequences and the Tms of the primers and probes used in the 16S sepsis multiplex assay. The *Candida *sp. and control primers and probes were the same as those used in the gene specific version of the assay [42].  Table 4 lists the bacterial and fungal genomic DNA targets of our pathogen test panel. 

### 2.2. 16S Assay Primer and Probe Design

The 16S sepsis multiplex assay presented an alternative to the archetypal gene specific target approach [42]. The alternating conserved and highly variable regions of the 16S rDNA in bacteria made it possible to amplify the gene in many different species of bacteria while still differentiating between them. These highly conserved regions served as templates for primer design, ensuring that all bacteria of interest would be amplified. The variable region that spanned the sequence between primers was used for identification and differentiation between species using Lights-On/Lights-Off probes in the Quasar 670 fluorescent dye channel. 

The Lights-On/Lights-Off probes were designed as consensus probes to bind to as many targets as possible with four On/Off pairs fully coating the region between the primers. The difference in binding Tm of probes to targets allowed for distinction between the 13 bacterial species that were included in our test panel. The output fluorescent composite of all probes rose and fell with temperature and yielded a fluorescent contour that was specific to a pathogen. 

Additional specific primer and probe sets were required for the *Candida* and internal control detection. The four *Candida* pathogens of our test panel were analyzed with the separate Cal Red 610 dye channel using specific primers and probes. A region of the P450L1A1 gene was used to create two pairs of specific primers made flexible through mis-matches to amplify the four *Candida* species. Two probes were designed in a similar manner to pick up only the *Candida* species of interest. In addition to the pathogenic targets in the test panel, specific primers and a probe were designed to amplify an unspecified gene of *Lactococcus lactis* subsp. *cremoris* to serve as an internal control detected in Cal Orange 560. Primer and probe sequences were designed for the LATE-PCR assay based on criteria previously described [36]. 

The primer and probe sequences were put through a series of bioinformatics analyses. The sequences were put through a Basic Local Alignment Search Tools (BLAST) search for comparison to all other sequences in the NCBI Genbank to ensure that there were sufficient nucleotide differences between the target organism and related, non-target organisms (near neighbors). Once the target-specific primers and probes passed the BLAST search evaluation, the monoplex assays were optimized against pathogen genomic DNA targets. After the validation of the monoplex assays, they were combined into a single tube multiplex assay, tested against all pathogen genomic DNA targets and all primer and probe sets. 

Table 5 lists the reagents and their concentrations used in the 16S sepsis multiplex assay. The thermal profile used on the Bio Rad IQ5 system for amplification and detection of the assay is as follows: 95°C for 3 min, followed by, 95°C/10 s, 65°C/15 s, 72°C/45 s for 45 cycles followed by a melt starting at 25°C with 1°C/ 30 s increases up to 80°C, followed by, an annealing starting at 80°C with 1°C/ 30 s decreases down to 25°C. Fluorescence was measured in the Cal Orange 560, Cal Red 610 and Quasar 670 channels at each anneal step. All data used in the analysis were taken from the final anneal curve.

### 2.3. 16S Sepsis Assay: Data Analysis and Making a Clinical Call

The final anneal fluorescence data were exported from the BioRad IQ5 2.1 software into Microsoft Excel. The anneal data were normalized to 75°C to place all of the amplification wells on the same level of background fluorescence and then the no template control (NTC) background was subtracted to get the normalized fluorescence contours. Subsequently, the highest fluorescent peak was then normalized to 1.0 to further put all fluorescence contours on the same scale. We have considered a number of approaches to data analyses that will allow a positive or negative call to be made for a specific pathogen within the error limits of the assay. Here we outline the best way for a software program to make a clinical call. An unknown sample will be amplified by the 16S sepsis multiplex assay and a fixed number of endpoint anneal readings will be taken after the amplification is complete and the probe binding has reached equilibrium, for instance a read every 5°C. The data would then be normalized as indicated above.

The fluorescence values at the temperature points can then be compiled into a table, through which the software can scan. The first scan done by the software determines at which temperature the sample has a value of 1.0. This temperature will correspond to one or more targets in a previously characterized library. Once that temperature is determined and the possible targets narrowed down, the fluorescence level at the temperature point above or below is then compared to the possible targets. This second comparison allows for the determination of the correct bacterial target because of the specific nature of the fluorescent contours. 

## 3. Results

The LATE-PCR 16S sepsis multiplex assay successfully identified all 13 bacterial and four fungal targets in our pathogen test panel in addition to an internal bacterial control in less than three hours. The assay also detects a mix of Gram positive and Gram negative bacterial species as well as fungal *Candida* species. This is important because sepsis can be due to a wide variety of pathogens. Because of the design of our Lights-On/Lights-Off probes, this assay will also identify bacterial species not included in our test panel.

### 3.1. LATE-PCR 16S Multiplex Assay

The assay configuration in Table 1 shows the distribution of the bacterial and fungal pathogens of the test panel listed in Table 4. All 13 bacterial targets were detected in a single color using the Lights-On/Lights-Off probe technology. Two remaining channels were used for fungal and internal control detection. Due to the bacterial nature of the control it also gave a fluorescent contour in the Quasar channel along with the other bacterial targets.

The primers were placed in two highly conserved regions of the 16S rDNA that flank a highly variable region (V3). These primers would, theoretically, amplify 98% of the >900,000 16S ribosomal DNA sequences in the Michigan State University's Ribosomal database [44]. For detection of multiple bacterial pathogens there was no wild-type sequence against which design fluorescent probes, thus consensus probes were the ideal option. The variable region was coated with 4 pairs of Lights-On/Lights-Off consensus probes. The differences in the DNA target sequence for each probe causes each probe/target Tm to vary and this, in turn, results in a unique contour for the set of probes to each bacterial pathogen target.

**Figure 2:**
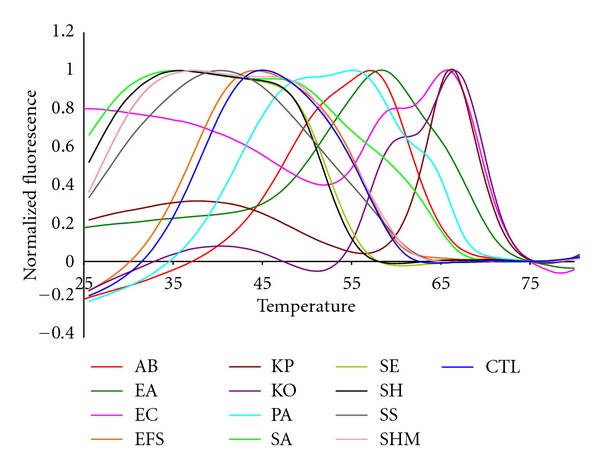
The normalized anneal curve showing all 16S targets in the Quasar channel with their highest peak normalized to 1.0.

Figure 2 shows the fluorescent contours of the 13 bacterial targets (referred to according to the abbreviations listed in Table 4). Each fluorescent contour in Figure 2 is the average of three replicate samples. We have previously shown that all three replicates are essentially identical within experimental limits [43]. The EFM was not shown because it gave the same contour as the EFS. This was true despite the fact that the sequences of the two amplicons differ by six bases. But, these sequence differences are not detected because none of the consensus probes targeted to these portions of the two amplicons has a Tm above 25°C. In fact, only three of the eight consensus Lights-On/Lights-Off probes in the set hybridized to these two *Enterococcus *targets, and the sequence under those three probes yielded the same fluorescent contours. Several possible remedies for this ambiguity are discussed below. 

**Figure 3:**
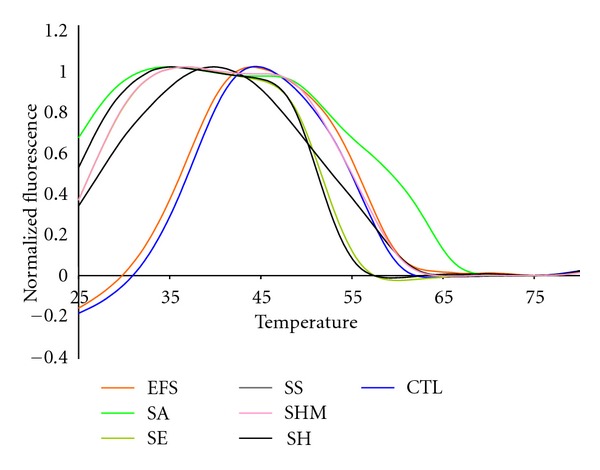
The normalized anneal curve showing all Gram positive 16S targets in the Quasar channel with their highest peak normalized to 1.0.

**Figure 4:**
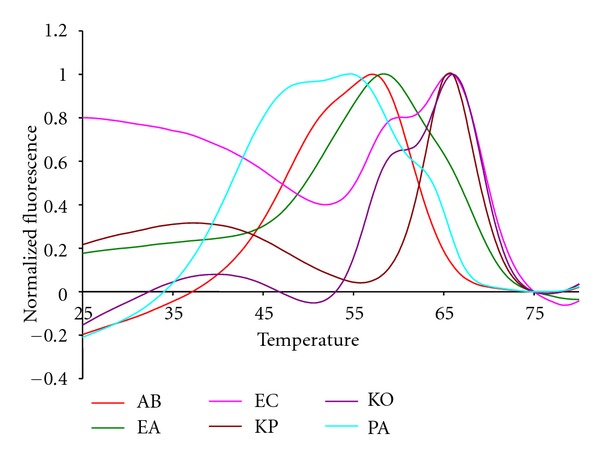
The normalized anneal curve showing all Gram negative 16S targets in the Quasar channel with their highest peak normalized to 1.0.

As shown by Figures 3 and 4, the bacterial targets of our test panel also segregated in temperature space on the basis of whether they are Gram positive or Gram negative bacteria. The relatively high GC content of the Gram negative species caused the Tm of their highest normalized fluorescent contours to be above 50°C. The fluorescent contours of the Gram positive bacteria, in contrast, are below 50°C. Figure 3 shows the fluorescent contours of the Gram positive targets. While several contours looked similar there were important nuances in the curves that allowed them to be distinguished from one another. The high reproducibility of the 16S sepsis multiplex assay and Lights-On/Lights-Off probes regardless of the starting concentration of genomic DNA means that these slight nuances are consistent and can be reliably used for species identification. The SA and EFS were the most different from most of the other targets, although the EFS and the CTL had similar contours.

**Figure 5:**
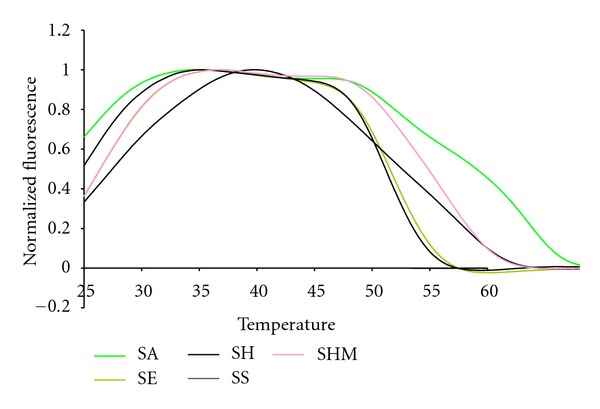
The normalized anneal curve showing the coagulase negative  16S targets in the Quasar channel with their highest peak normalized to 1.0.

 Several Staphylococcal species were among the Gram positive species tested, Figure 5. The fluorescent contours of SHM and SS are distinguishable from each other and from the SE and SH  contours between 45°C and 50°C, although SE and SH are not distinguishable from each other at that temperature. But SE and SH are clearly distinguishable from each other between 25°C and 30°C. These results illustrate that multiple pairs of Lights-On/Lights-Off probes generate a high level of information because they test probe/target interactions over a wide temperature space.

Figure 4 shows the fluorescent contours for the Gram negative targets of the test panel. As with the Gram positive targets these contours are highly reproducible, regardless of the starting concentration of genomic DNA. The KO and KP species are distinct from one another because the KO has an extra shoulder indicating the binding of a Lights-On probe at a higher temperature, 60°C, than the binding of its paired Lights-Off probe at 55°C. The two *Enterobacter* species can also be distinguished because the Lights-On signal arising from EA remains fluorescent down to a lower temperature than the EC signal. The paired Lights-Off probe binds to EA at about 53°C and to EC at about 63°C. 

In order to test the limit of detection for each bacterial species, genomic DNA was diluted from 100,000 bacterial genomes down to 10 genomes in tenfold steps.  Figure 8 depicts a representative dilution series for SE. All samples were amplified through a total of 45 thermal cycles. Because the 16S rDNA is present from 1 to 15 copies in some bacterial genomes [45], we regard detection at the 10 copy level as indicative of as little as a single bacterial genome present initially in the 25 *μ*L sample. Regardless of how many genomes were present initially all samples exhibit the same fluorescent contours when the highest peak present after 45 cycles is used to normalize the sample across the entire detection temperature range. This result once again confirms that fluorescent contours and fluorescent signatures are highly reproducible features of LATE-PCR analysis with Lights-On/Light-OFF probes [43].

**Figure 6:**
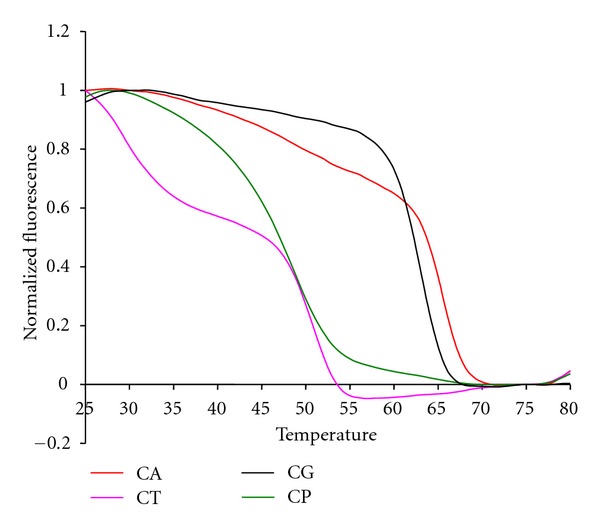
The anneal curve showing the fluorescent contour of the four tested *Candida* species in the Cal Red channel.

**Figure 7:**
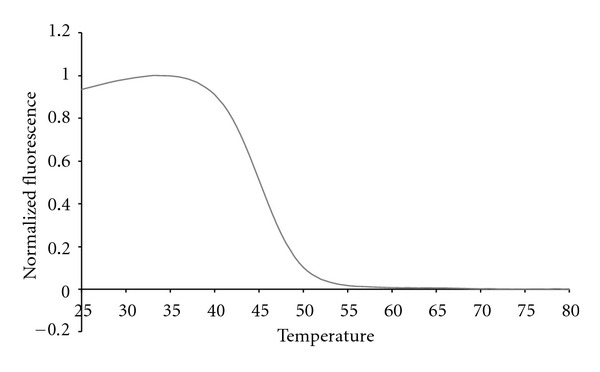
The anneal curve showing the fluorescent contour of the control *L. lactis* in the Cal Orange channel.

**Figure 8:**
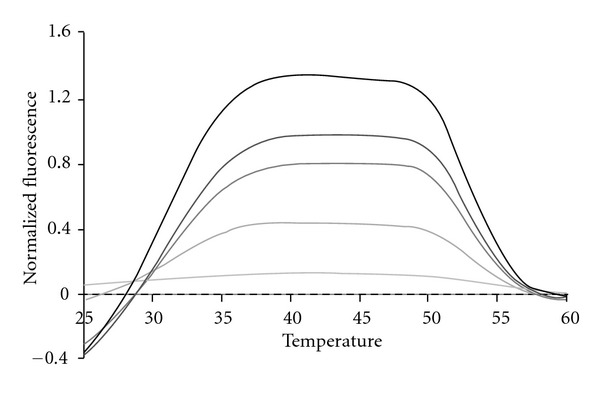
The fluorescent contour of a SE dilution series from 10^5^ genome copies down to 10 genome copies as shown by the gradient from dark (10^5^) to light (10). The subtracted NTC background is shown by the dashed line to emphasize that the 10 copy dilution is above background.

The complete multiplex assay described here also used Cal Red 610 probes (Figure 6) to test for the presence of 10^5^ copies of genomic DNA from *Candida *sp. and Cal Orange 560 probes (Figure 7) to test for 10^5^ copies of control genomic DNA from *Lactococcus lactis*. 

The 16S sepsis multiplex assay resolved four different *Candida* species using two pairs of primers to amplify a region of the P450L1A1 gene and two sets of probes to analyze the single-stranded products. The first set of primers amplified three of the four species, *C. albicans, C. parapsilosis*, and* C. tropicalis.* The second set of primers amplified both *C. glabratta *and *C. krusei, *although the latter species was not actually tested in experiments reported here. The probe sets were designed to generate unique signals for each of the four possible species. These probes were molecular beacon probes and therefore exhibited unique melting temperatures rather than fluorescent contours. 

The internal control sequence from *L. lactis* was used to ensure that the sample preparation protocol did not compromise the integrity of the genomic DNA from any of the bacteria being tested. This sequence was amplified using its own set of primers and detected using a Cal Orange probe. But *L. lactis *genomic DNA also generated a 16S fluorescent contour in the Quasar 670 channel because it is a bacterial species. In order to avoid this ambiguity in the future we are currently investigating other non-bacterial controls that do not possess a 16S gene target, but still accurately assess the sample preparation protocol. 

### 3.2. Mixtures of Bacterial Targets

**Figure 9:**
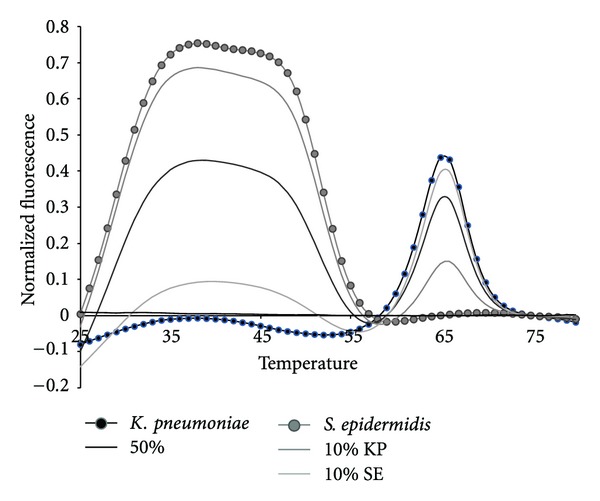
The fluorescent contours of mixtures of *K. pneumoniae and S. epidermidis *in the Quasar channel.

**Figure 10:**
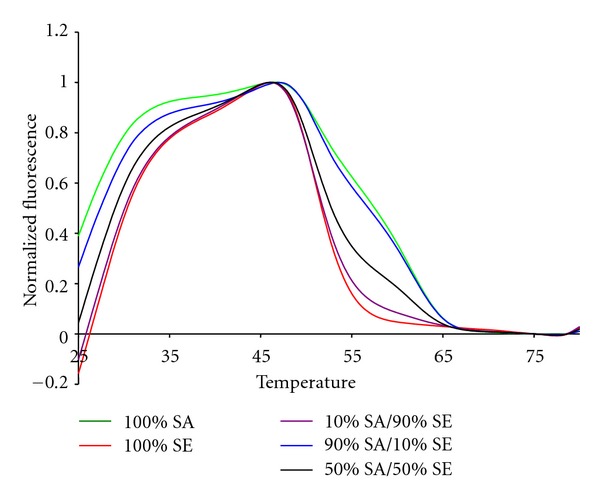
The fluorescent contours of mixtures of *S. aureus* and *S. epidermidis* in the Quasar channel.

The 16S sepsis assay has to detect the presence of more than one bacterial pathogen. Therefore we tested the 16S sepsis multiplex assay against mixtures of targets, specifically focusing on sepsis panel targets versus SE.  Figure 9 shows experimental data for a mixture of 10% KP : 90% SE, 50% KP and SE, and 10% SE : 90% KP with pure KP and SE also shown.  Figure 10 shows the normalized experimental data for 10% SE : 90% SA, 50% SA and SE, and 10% SA : 90% SE mixtures as well as pure SA and pure SE shown.

All target mixtures had 10,000 copies of total genomic DNA. Mixtures of 50 : 50, 10 : 90, and 1 : 99 were assessed. But, the 1 : 99 mixtures did not differ from the NTC controls by more than three standard deviations and are not shown. The two sets of mixtures shown here illustrate several interesting features of the analytical method. The fluorescent contours for KP and SE do not overlap in temperature space. Thus mixtures of these two organisms demonstrate the greatest possible sensitivity for either minor component, Figure 9. This combination of organisms also represents a mixture of a Gram positive and Gram negative species. The data in Figure 9 show that at least 10% of one pathogen in the presence of 90% of the other can be distinguished from pure samples. 

In contrast, SA and SE have very similar fluorescent contours. The data in Figure 10 show that 50 : 50 mixtures of these two Gram positive species can readily be distinguished from pure samples. The 10 : 90 and 90 : 10 mixtures are also resolvable, but have fluorescent contours are similar to the 100% contours. 

### 3.3. 16S Sepsis Assay: Background Human DNA

**Figure 11:**
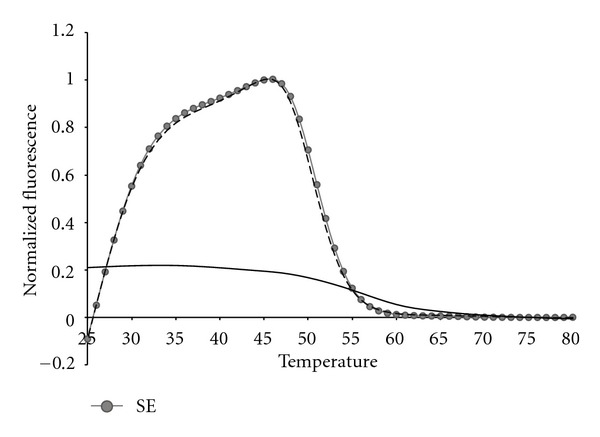
The dashed line shows the fluorescent contour for S. epidermidis plus 35,000 copies of human genomic DNA. As shown, the SE contour is not impacted by addition of human gDNA as it matches the contour for SE alone (-°-). The contour for human gDNA is shown by the solid black line.

The presence of human genomic DNA is yet another possible challenge to the robustness of any assay for detection of bacterial 16S genomic DNA. Figure 11 shows the fluorescent contour of a mixture of 10^5^ copies of SE genomic DNA plus 35,000 copies of Promega Human genomic DNA, as compared to pure SE, or pure human genomic DNA separately. It is clear that the human DNA has no effect on the bacterial DNA fluorescent contour.

## 4. Discussion

Most molecular diagnostic assays make use of symmetric PCR for target amplification and sequence-specific fluorescent probes for real-time detection of the double-stranded DNA products. These NAT based technologies have thus far not lived up to their promise because they lack the capacity to provide all of the needed information in a single-closed tube reaction. As a result, a sepsis sample has to have high numbers of pathogens so that it can be split and run in several tubes in parallel. The solution to this problem lies in use of a single-tube reaction that yields accurate, reliable, relevant information from a sample having low pathogen concentration. The LATE-PCR Sepsis Assay described here begins to address several of these goals. 

An alternative, gene specific version LATE-PCR assay described elsewhere is also highly informative and very sensitive [42]. When looking to identify particular pathogens, the gene specific assay is capable of detecting down to a single genome copy of the gene even in the presence of other bacterial targets. The gene specific assay is been tested in coinfection scenarios in a human DNA background with a reliable detection ratio of 99 : 1. The specificity and sensitivity levels of the gene specific assay made it highly useful for reliable detection of pathogens in a clinical setting. The limitation of the gene specific assay is that it cannot detect a pathogen for which it is not designed. For this reason the 16S rDNA LATE-PCR assay described here provides greater coverage than the gene specific assay. 

The 16S rDNA sepsis multiplex assay achieves broad coverage by making use of consensus probes together with our novel Lights-On/Lights-Off probe technology. These two components are synergistic, and each can be expanded further to achieve even broader pathogen detection and identification. The primers are to conserved sequences and are therefore nearly universal in their potential to amplify any bacterial 16S gene target. Indeed, the high sensitivity of the primers actually introduces a potential unique limitation of the assay, since it detects *E. coli *genomic DNA present in many commercial Taq polymerases. As a result the polymerase enzyme has to be highly purified without traces of *E. coli* rDNA in the background.

The 16S assay is also readily expandable. The additional underutilized fluorescent dye channels can be used to include more probe sets for additional pathogen detection. These probe sets provide further distinction between the increased numbers of targets. For example, we have been able to completely differentiate EFS and EFM fluorescent contours by adding an additional set of Lights-On/Lights-Off probes in the Cal Red 610 channel (data not shown).

Other sepsis molecular diagnostic assays make use of the 16S rDNA target for amplification of multiple pathogens. The SepsiTest, for instance, uses the 16S gene to amplify any bacterial target present in the sample, but the pathogens were not readily distinguished until the amplified products were sequenced [46, 47]. Sequencing adds cost and time to diagnosis, and might result in laboratory contamination from released amplicons. In contrast, the LATE-PCR 16S sepsis multiplex assay described here amplifies and identifies many pathogens in the same closed tube. Only unrecognized pathogens need be sequenced, and once sequenced their unique fluorescent contours are immediately recognizable in future tests.

The SeptiFast Light Cycler assay is able to detect and identify 25 common sepsis pathogens in a short time frame without using sequencing [48]. But, the SeptiFast Light Cycler assay depends on splitting a 50 *μ*L sample into three parallel tubes to identify Gram positive bacteria, Gram negative bacteria, and fungi. The LATE-PCR 16S assay, in contrast, is a triplex assay that makes the same calls in a single-tube having a total reaction volume of just 25 *μ*L.

## 5. Conclusions

The LATE-PCR 16S sepsis multiplex assay described here reliably detected 13 bacterial species and four *Candida* species and gave consistent fluorescent contours for the infectious targets of our test panel. We have shown the assay detected down to 10 genome copies, as well as differentiated as little as a 10% mixture when maximizing temperature space. The technology employed to construct this assay is novel and highly flexible.

## Figures and Tables

**Table 1:** 16S sepsis multiplex assay configuration.

Temperature	Cal Orange	Cal Red	Quasar
High ↓ Low	*L. lactis *	*C. albicans *	16S Bacterial Targets
		*C. glabratta *	
		*C. parapsilosis *	
		*C. tropicalis *	

**Table 2:** 16S On/Off Predicted Probe Tms °C.

Target	Quasar 1 On Probe	Quasar 2 On Probe	Quasar 3 On Probe	Quasar 4 On Probe	Quasar 1 Off Probe	Quasar 2 Off Probe	Quasar 3 Off Probe	Quasar 4 Off Probe
AB	50.2	64.9	18.1	59.5	50.8	42.4	18.3	40.3
EA	69.5	58.6	11.6	69.7	61.5	54.5	18.3	42.1
EC	69.5	58.6	11.6	69.7	61.5	54.5	33	56.4
EFS	24.1	59.7	−2.8	70.6	7.8	34.6	20.6	−3.2
EFM	24.1	59.7	−2.8	70.6	7.8	34.6	20.6	−3.2
KP	70.6	66	13.8	70.6	62.6	64.5	35	57.7
KO	69.5	58.6	11.6	69.7	61.5	54.5	33	56.4
PA	68.9	66	−2.8	58	52	60	35	39.6
SA	54.5	53.8	63.9	34.7	36.8	39.3	20.6	30.3
SE	54.5	53.8	53.9	34.7	36.8	39.3	20.6	30.3
SH	54.5	53.8	50.1	34.7	36.8	38	20.6	30.3
SS	54.5	53.8	58.2	34.7	36.8	0.9	20.6	30.3
SHM	54.5	53.8	56.7	34.7	36.8	38	20.6	30.3

**Table 3:** LATE-PCR 16S sepsis multiplex primer and probe sequences.

Primer	Sequence 5′→3′	Bases
16S limiting primer	CCAGACTCCTACGGGAGGCAGCAGT	25
16S excess primer	GTATTACCGCGGCTGTGGCA	20
*Candida* limiting Primer APT	ACCATTACCTCATTATTGGAGACGTGATGCTGC	33
*Candida* excess Primer APT	GCAATTTCTTGATCAGTCATTTTTACACCATCTT	34
*Candida * limiting Primer G	ATGCCCAACAAGCTATCTCTGGTACTTACATGT	33
*Candida* excess Primer G	CGGATGTTGCAGGGGAAGTATGTTGACCACCCA	33
*L. lactis *(control) Limiting Primer	CTAAAATCAGGAACTTCGTTATCTTTAGTAGTCACAACCA	40
*L. lactis *(control) Excess Primer	TAATCATTATTCCTCAAGAAGAGATACAATCGGTCA	36
Control probe	Cal Org 560-ATAAACCTTTCTTAAAAT-BHQ1	18
*Candida* APT probe	Cal Red 610-ATGTGATATTGATCCAAATCGTGATTTAATAT-BHQ2	32
*Candida* G probe	Quasar 670-AAACAAGGATGGTACTAGGATGACCGTT-BHQ-2	28
16S Quasar 1 On	Quasar 670-AAGCGAAAGCCTGATGCAGCCATT-BHQ2	24
16S Quasar 2 On	BHQ2-TAGCCGCGTGTGTGAAGAATA-Quasar 670	21
16S Quasar 3 On	Quasar 670-TTATATGTGTAAGTAACTGTGCACATCAA-BHQ2	29
16S Quasar 4 On	Quasar 670-TTGAAGAAGCACCGGCTAACTCCGAA-BHQ2	27
16S Quasar 1 Off	AAGGGGAATATTGCACAATGGTT-BHQ2	23
16S Quasar 2 Off	BHQ2-TTGGCCTTCGGATTGTAAAGCACTTAA-C3 Carbon Linker	27
16S Quasar 3 Off	TATTAGTAGGGAGGAAGTA-BHQ2	19
16S Quasar 4 Off	TTGACGTTACCCGCAA-BHQ2	16

**Table 4:** Bacterial and fungal genomic DNA targets for 16S sepsis multiplex assay.

Target	Abbreviation	Accession ID	ATCC Acquisition
Bacteria			
* A. baumannii *	AB	NC_010400	17978D-5
* E. aerogenes *	EA	HM480361	15038D-5
* E. cloacae *	EC	GU979185.1	13047D-5
* E. faecalis *	EFS	NC_004668	700802D-5
* E. faecium *	EFM	AJ301830	BAA-472D-5
* K. pneumoniae *	KP	NC_011283	BAA-1706D-5
* K. oxytoca *	KO	NR_041749.1	Received from UC Davis
* P. aeruginosa *	PA	NC_009656	17933D
* S. aureus *	SA	NC_002745	Barry Kreiswirth PHRI
* S. epidermidis *	SE	NC_002976	12228D-5
* S. haemolyticus *	SH	X66100	29970D-5
* S. saprophyticus *	SS	EF522127.1	Received from UC Davis
* S. hominis *	SHM	X66101	Received from UC Davis
* L. lactis *(Control)	CTL	NC_008527	Received from Smiths
Fungi			
* C. albicans *	CA	AF153846	14053D-5
* C. parapsilosis *	CP	GQ302972	Received from UC Davis
* C. tropicalis *	CT	AY942643	Received from UC Davis
* C. glabratta *	CG	AY942647	Received from UC Davis

**Table 5:** LATE-PCR 16S sepsis multiplex reagents.

Starting Concentration	Reagent	Final Concentration	Amt/25 uL reaction	Manufacturer
10x	PCR buffer	10 mmol^−1^	In beads	GE healthcare
10 mmol^−1^	dNTPs	200 umol^−1^	In beads	GE healthcare
50 mmol^−1^	Mg^++^	1.63 mmol^−1^	1.13 uL + beads	Invitrogen
10 umol^−1^	Limiting Primer (4)	50 nmol^−1^	0.125 uL	Sigma
100 umol^−1^	Excess Primer (4)	1 umol^−1^	0.250 uL	Sigma
10 mmol^−1^	On Probe (7)	100 nmol^−1^	0.250 uL	Biosearch
10 mmol^−1^	Off Probe (4)	300 nmol^−1^	0.750 uL	Biosearch
2.5 U/Bead	Pure Taq ready-to-go beads	1.25 U	0.5 bead	GE healthcare
10^6^ genome copies	gDNA	10^5^ genome copies	1 uL	ATCC
